# Organ protection via core temperature control in Type A aortic dissection using pure hypothermia alone

**DOI:** 10.1186/s13019-026-03915-2

**Published:** 2026-02-28

**Authors:** Hodaka Wakisaka, Tomoaki Suzuki

**Affiliations:** Department of Cardiovascular Surgery, Shiga University of Medical Science, Setatsukinowa, Otsu , 520-2192 Shiga Japan

**Keywords:** acute type A aortic dissection, organ protection, hypothermic circulatory arrest, core temperature

## Abstract

**Background:**

A new consensus on temperature for organ protective strategies during circulatory arrest on aortic surgery was established in the 2024 EACTS/STS Guidelines. The aim of this study is to evaluate early postoperative outcomes in patients undergoing hemiarch replacement for acute type A aortic dissection (TAAD) using hypothermic circulatory arrest without adjunctive cerebral perfusion, stratified according to guideline-based core temperature categories.

**Methods:**

The 358 patients who underwent surgical hemiarch replacement with hypothermic circulatory arrest, no cerebral perfusion, as organ protection for TAAD at Shiga University of Medical Science between August 2012 and August 2022 were included in this study. Patients were classified by nadir core temperature during circulatory arrest into low–moderate hypothermia (20.1–24.0 °C; LMH, *n* = 30), high–moderate hypothermia (24.1–28.0 °C; HMH, *n* = 212), and mild hypothermia (≥ 28.1 °C; MH, *n* = 116). Early clinical outcomes were compared using multivariable adjustment and restricted cubic spline analyses.

**Results:**

The overall stroke rate was 11.7% and the 30-day mortality rate was 6.4%. After adjustment, the rate of stroke and reoperation for bleeding was significantly higher in the LMH group (*p* = 0.002, 0.001; reference to MH group). Restricted cubic spline analysis showed a non-linear association between nadir core temperature and stroke, with higher adjusted risk ratios observed at temperatures below approximately 27 °C. These associations were observed in the context of baseline imbalances, including a higher prevalence of carotid malperfusion in lower temperature groups.

**Conclusion:**

In patients undergoing hemiarch replacement without cerebral perfusion, a greater reduction in nadir core temperature was not associated with improved neurological outcomes when core temperature was used as an indicator. The observed association between lower core temperature and adverse outcomes may reflect residual confounding related to preoperative or intraoperative malperfusion rather than a direct causal effect of temperature itself. These findings suggest that core temperature alone may be an insufficient surrogate for brain protection and should be interpreted cautiously in the evaluation of neurological risk.

**Supplementary Information:**

The online version contains supplementary material available at 10.1186/s13019-026-03915-2.

## Introduction

On surgery for acute type A aortic dissection (TAAD), organ protection, specifically brain protection, during circulatory arrest is essential, affecting postoperative outcomes such as survival and neurological complications. However, the optimal strategy as methods and temperature of organ protection for TAAD remains controversial. One of the reasons is that there have been no clear temperature criteria. There have been many discussions on the optimal temperature for circulation arrest, primarily based on the temperature definition (pharyngeal temperature) defined by Yan et al. [1–3]. However, in the 2024 EACTS/STS Guidelines, considering the core temperature (bladder or rectal) as the reference value for the determination of the level of lower body hypothermia circulatory arrest (HCA) is recommended, and the categories were subdivided [4]. It has changed from pharyngeal temperature, which reflects more central temperatures, to core temperature because it is questioned whether the spinal cord and the other organs are protected similarly, especially during more extended periods of circulatory arrest. The guidelines recommend monitoring nasopharyngeal and core temperatures (class Ⅰ, level C); however, recent studies on organ protection are increasingly based on core temperature [5–8]. It remains uncertain whether it is appropriate to standardize core temperature as an indicator when discussing brain protection. Nevertheless, recent studies based on core temperature also discuss brain complications, including stroke, with the same evaluation as other organ complications. These reports are generally based on cerebral protection combined with hypothermia and cerebral perfusion. In our perspective, the reason why brain temperature has not been emphasized is that cerebral perfusion is generally assumed to be provided. Furthermore, the appropriate temperature during HCA even for brain protection is discussed based on core temperature. We believe that this issue warrants further evaluation, particularly in settings without adjunctive cerebral perfusion. In our institutional practice, cerebral perfusion is not routinely employed for selected hemiarch procedures; therefore, this cohort provides a unique opportunity to evaluate the role of hypothermia and temperature indicators without the confounding effects of active cerebral perfusion.

This study aimed to evaluate the relationship between core temperature and organ protection in a new consensus, with a focus on organ protection through lower body temperatures due to the absence of brain perfusion, and to examine whether core temperature is an appropriate indicator for the evaluation of brain protection.

## Subjects

**Fig. 1:**
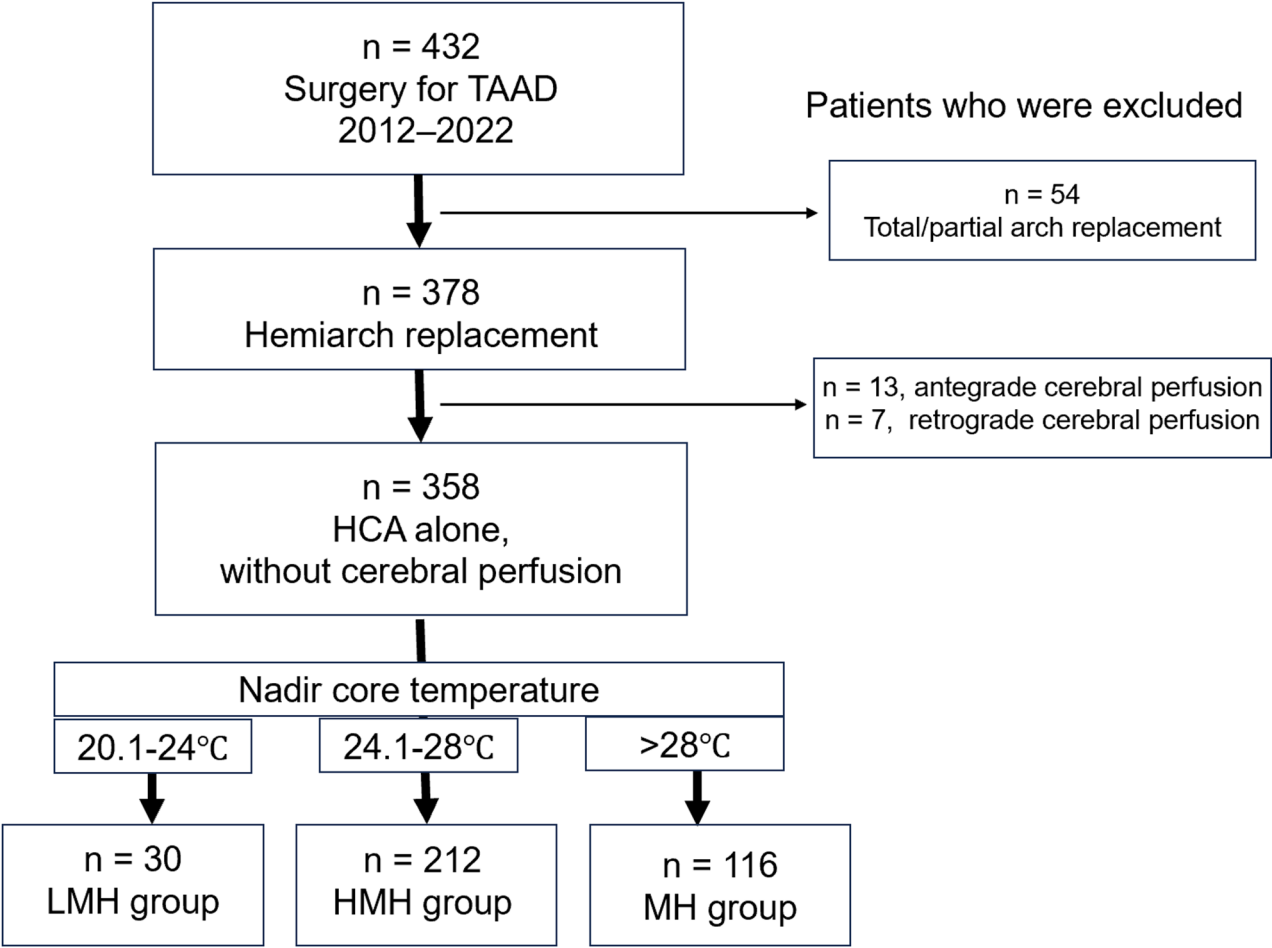
Patient selection. TAAD, acute type A aortic dissection; HCA, hypothermic circulatory arrest; LMH, low moderate hypothermia; HMH, high moderate hypothermia; MH, mild hypothermia

We performed a retrospective analysis of a database from August 2012 to August 2022 to identify patients who underwent hemiarch replacement with HCA without cerebral perfusion for TAAD at Shiga University of Medical Science. The data used in this study were extracted from the medical records. 432 patients received emergency surgical treatment for TAAD during this period. Among them, 378 underwent hemiarch replacement, and 54 had total or partial arch replacements. After excluding 13 patients who used antegrade cerebral perfusion (ACP) and 7 who had retrograde cerebral perfusion (RCP), 358 patients were deemed eligible for inclusion in this study. (Fig. 1) The study was approved by the Institutional Review Board of Shiga University of Medical Science (approval number R2021-167) on February 15, 2022. Given the retrospective design of the study, the requirement for written informed consent was waived. Identifiers that could potentially identify participants were removed from the data to ensure confidentiality.

## Methods

### Outcome measures and definitions

The primary outcome was postoperative stroke. The secondary outcomes included early mortality and other postoperative complications.

According to the 2024 EACTS/STS guidelines, they were stratified into three groups based on their nadir core temperature during circulatory arrest. Those groups are the low moderate hypothermia (20.1 °C–24℃, LMH group, *n* = 30), high moderate hypothermia(24.1 °C–28℃, HMH group, *n* = 212) and mild hypothermia(≥ 28.1℃, MH group, *n* = 116) groups.

Postoperative stroke was defined as a persistent neurological deficit, with evidence of cerebral infarction on computed tomography or magnetic resonance imaging, as confirmed by a neurologist. Preoperative conditions were not taken into account, and the onset was considered to occur at any point during the perioperative period. Additionally, patients who exhibited clear signs of severe cerebral damage or edema and died without undergoing a postoperative neurological assessment were also classified as having experienced a postoperative stroke. Hemodynamic instability was defined by preoperative hypotension (systolic blood pressure < 60 mmHg), which could be caused by factors such as bleeding, cardiac tamponade, malperfusion, myocardial ischemia or infarction, or acute congestive heart failure. Malperfusion was defined by preoperative imaging findings showing reduced blood flow to the various organs associated with dissection and the appearance of organ dysfunction.

Core temperature was basically applied to bladder temperature, but as an exception, rectal temperature was applied to anuric patients on long-term hemodialysis. The definitions for the other components were based on the STS-ACSD criteria [9].

### Surgical technique

Previous studies have outlined the surgical technique for TAAD at our institution [10].

In brief, our strategy is to decide whether to do hemiarch replacement or total arch replacement based on the entry position. If an intimal tear was found in the ascending aorta or proximal transverse aorta, or if no tear was present in the transverse aorta upon direct visualization, only hemiarch replacement was performed. Median thoracotomy and cardiopulmonary bypass (CPB) were universally performed on all patients. The strategy of organ protection is to start whole-body cooling after vent tube insertion, with a tympanic temperature of 25 °C as a guideline for circulation arrest. In this case, if the core temperature has not dropped below 30 °C, cooling is continued until it does, but the core temperature itself is not used as a guide for circulation arrest. The main characteristic of our organ protection strategy is that hypothermia alone was used during circulatory arrest in selected hemiarch procedures, without routine use of cerebral perfusion. As an exception, ACP or RCP was performed if cerebral oxygenation, monitored with the INVOS 5100 C oximeter (Somanetics, Troy, MI, USA), significantly declined or if the complexity of distal anastomosis was expected to result in a circulatory arrest lasting more than 30 min. The decision to perform hemiarch or total/partial arch replacement, as well as whether to include cerebral perfusion, was left to the surgeon’s judgment.

### Statistical analysis

Stratification was performed based on the core temperature at circulation arrest and divided into three groups. Categorical data were expressed using counts and percentages compared using χ², and Fisher’s exact tests for frequencies of < 5. Continuous variables were presented as means with standard deviation or medians with interquartile ranges. As all continuous variables had skewed distributions assessed using the Kolmogorov–Smirnov test, Kruskal–Wallis tests were employed when compared.

Modified Poisson regression was used to estimate the risk of outcomes, including 30-day mortality, stroke, paralysis, and bleeding [11]. The primary exposure variable was the nadir core temperature categories. Risk ratios (RR) with 95% confidence intervals (CI) for the LMH and HMH groups were calculated using the MH group as the reference. Crude and adjusted models were applied, with adjusted models incorporating stabilized weights based on propensity scores.

Propensity scores were derived using multinomial logistic regression with core temperature as the response variable and confounders as explanatory variables. Confounders included preoperative characteristics such as age, body mass index, sex, history of cerebrovascular accident, renal insufficiency, malperfusion of each organ (coronary, carotid, visceral and renal, limbs), neurological deficits, hemodynamic instability, cardiopulmonary resuscitation and intraoperative factors such as cardiopulmonary bypass time, circulatory arrest time because they have a significant impact on postoperative outcomes.

Stabilized weights were calculated as the marginal probability of core temperature divided by the propensity score and applied after confirming standard support across groups. Balance was assessed using absolute standardized mean differences (< 0.1) and variance ratios (0.8–1.25) (Supplement 1). Residual imbalance in several covariates was anticipated given the observational nature of the study and was further considered in the interpretation of the results. Restricted Cubic Spline (RCS) analysis with three knots (10th, 50th, and 90th percentiles) was conducted to explore the association between core temperature and stroke risk [12]. Stabilized weights were used for confounding adjustment, as in the modified Poisson regression. A nadir core temperature of 27 °C was selected as the reference value for RR for descriptive purposes. The spline knots were set at 24.4 °C, 27.2 °C, and 29.6 °C.

Statistical analysis and data visualization were using R 4.3.1 and Microsoft Excel v2402. A P value of < 0.05 indicated statistically significant.

## Results

### Characteristics of the patients

In total, 358 patients underwent hemiarch repair with HCA without cerebral perfusion as a brain-protective strategy for TAAD from 2012 to 2022. Table 1 summarizes the baseline characteristics of the three groups. The mean patient age was 69.3 (12.9) years, with fewer elderly patients in the MH group (*p* = 0.03). Patients in the LMH groups were more frequently diagnosed with preoperative renal failure and malperfusion of the carotid arteries (*p* = 0.03). This imbalance suggests a higher baseline disease severity in patients who reached lower nadir core temperatures. The other preoperative characteristics and dissection-related variables were generally comparable between the three groups.

**Table 1:** Preoperative characteristics of the patients

Characteristics	All	LMH	HMH	MH	*P* Value
	(*n* = 358)	(*n* = 30)	(*n* = 212)	(*n* = 116)	
Age, years, ±SD	69.3 ± 12.9	69.8 ± 14.6	70.7 ± 12.5	66.6 ± 12.6	0.02
≧80 years	90 (25.1)	8(26.7)	65(30.7)	17(14.7)	0.006
Body mass index, kg/m², ±SD	23.3 ± 4.0	22.4 ± 3.9	23.2 ± 3.9	23.8 ± 4.2	0.21
Male	195 (54.5)	18 (60)	111 (52.4)	66 (56.9)	0.6
Hypertension	243 (67.9)	19 (63.3)	144 (67.9)	80 (69.0)	0.84
Diabetes mellitus	34 (9.5)	3 (10.0)	17 (8.0)	14 (12.1)	0.49
Dyslipidemia	101 (28.2)	10 (33.3)	63 (29.7)	28 (24.1)	0.45
Smoking history	143 (39.9)	11 (36.7)	79 (37.3)	53 (45.7)	0.31
Old cerebrovascular accident	37 (10.3)	4 (13.3)	25 (11.8)	8 (6.9)	0.32
Marfan syndrome	17 (4.7)	2 (6.7)	10 (4.7)	5 (4.3)	0.86
Renal insufficiency	38 (10.6)	7 (23.3)	23 (10.8)	8 (6.9)	0.03
Lung disease	49 (13.7)	3 (10.0)	29 (13.7)	17 (14.7)	0.8
Previous cardiac surgery	11 (3.1)	1 (3.3)	8 (3.8)	2 (1.8)	0.59
DeBakey Type Ⅱ	72 (20.1)	4 (13.3)	46 (21.7)	22 (19.0)	0.53
Malperfusion	74 (20.1)	8 (26.7)	41 (19.3)	25 (21.6)	0.62
Coronary	11 (3.1)	1 (3.3)	6 (2.8)	4 (3.4)	0.95
Carotid	45 (12.6)	8 (26.7)	27 (12.7)	10 (8.6)	0.03
Visceral, renal	38 (10.6)	4 (13.3)	20 (9.4)	14 (12.1)	0.67
Extremities	30 (8.4)	1 (3.3)	16 (7.5)	13 (11.2)	0.3
Aortic valve insufficiency	44 (12.3)	6 (20.0)	26 (12.3)	12 (10.3)	0.36
Neurological deficit	67 (18.7)	6 (20.0)	36 (17.0)	25 (21.6)	0.59
Hemodynamic instability	51 (14.2)	8 (26.7)	26 (12.3)	17 (14.7)	0.11
Cardiopulmonary resuscitation	22 (6.1)	2 (6.7)	10 (4.8)	10 (8.6)	0.37

SD, standard deviation

### Operative and postoperative data

The operative data is summarized in Table 2. The cannulation site and concomitant procedures did not significantly among the groups. As expected, the more hypothermic the patient, the longer the operative and CPB, cross clamp times were.

**Table 2:** Operative data

	All	LMH	HMH	MH	*P* Value
	(*n* = 358)	(*n* = 30)	(*n* = 212)	(*n* = 116)	
Cannulation site					
Ascending aorta	22 (6.1)	3 (10.0)	14 (6.6)	5 (4.3)	0.47
Axillary artery	56 (15.6)	2 (6.7)	35 (16.5)	19 (16.4)	0.37
Femoral artery	272 (76.0)	25 (83.3)	159 (75.0)	88 (75.9)	0.61
Axillary་ Femoral artery	8 (2.2)	0	4 (1.9)	4 (3.4)	0.45
Duration of procedure, min, median(IQR)					
Operative time	175[156.3–201.8]	188.5 [176.8–218.8]	175 [158–201.5]	169 [152–196.5]	0.0068
Cardiopulmonary bypass time	97 [88–112]	109 [97–122.5]	100 [90–113]	93 [82.3–104.5]	< 0.001
Cross clamp time	46 [39–55]	48.5 [42–56.8]	48 [41–56.5]	44 [37.8–49]	0.0055
Circulatory arrest time	20 [17–23]	21.4 [18.2–24.1]	20.5 [17–23]	19.6 [17–22]	0.08
Nadir temperature, Celsius, median(IQR)					
Tympanic	24.5 [23.4–25.0]	24.2 [22.8–25]	24.6 [23.4–25]	24.5 [23.5–25]	0.53
Core (bladder or rectal)	27.2 [25.7–28.5]	22.5 [22.2–23.5]	26.6 [25.5–27.4]	29 [28.6–29.8]	< 0.001
Concomitant operation					
Aortic root replacement	5 (1.4)	1 (3.3)	2 (1.0)	2 (1.7)	0.54
Aortic valve repair or replacement	23 (6.4)	3 (10.0)	12 (5.7)	8 (6.9)	0.64
Coronary bypass	26 (7.3)	3 (10.0)	15 (7.1)	8 (6.9)	0.83
Lower limb bypass	16 (4.5)	2 (6.7)	9 (4.2)	5 (4.3)	0.83

IQR, interquartile range

Postoperative details are summarized in Table 3. The overall 30-day mortality was 23 (6.4%). Stroke, paraplegia, postoperative hemodialysis, and reoperation for bleeding were 42(11.7%), 8(2.2%), 24(6.7%), and 52(14.5%), respectively. Significantly more stroke and reoperation for bleeding in the LMH group [10(33.3%), 22(10.4%), 10(8.6%); *p* < 0.01, 12(40%), 28(13.2%), 12(10.3%); *p* < 0.01].

**Table 3:** Postoperative data

	All	LMH	HMH	MH	*P* Value
	(*n* = 358)	(*n* = 30)	(*n* = 212)	(*n* = 116)	
30-day mortality	23 (6.4)	2 (6.7)	12 (5.7)	9 (7.8)	0.76
Hospital mortality	24 (6.7)	3 (10)	11 (5.2)	10 (8.6)	0.37
Stroke	42 (11.7)	10 (33.3)	22 (10.4)	10 (8.6)	< 0.01
Spinal ischemia	8 (2.2)	2 (6.7)	2 (1.0)	4 (3.4)	0.08
Newly hemodialysis	24 (6.7)	4 (13.3)	11 (5.2)	9 (7.8)	0.21
Reoperation for bleeding	52 (14.5)	12 (40)	28 (13.2)	12 (10.3)	< 0.01
Tracheostomy	28 (7.8)	3 (10)	15 (7.1)	10 (8.6)	0.79
GI complication	12 (3.4)	2 (6.7)	6 (2.8)	4 (3.4)	0.55
Postoperative IABP, PCPS	9 (2.5)	1 (3.3)	5 (2.4)	3 (2.6)	0.95

GI, gastrointestinal; IABP, intraaortic balloon pumping; PCPS, percutaneous cardiopulmonary support

**Fig. 2:**
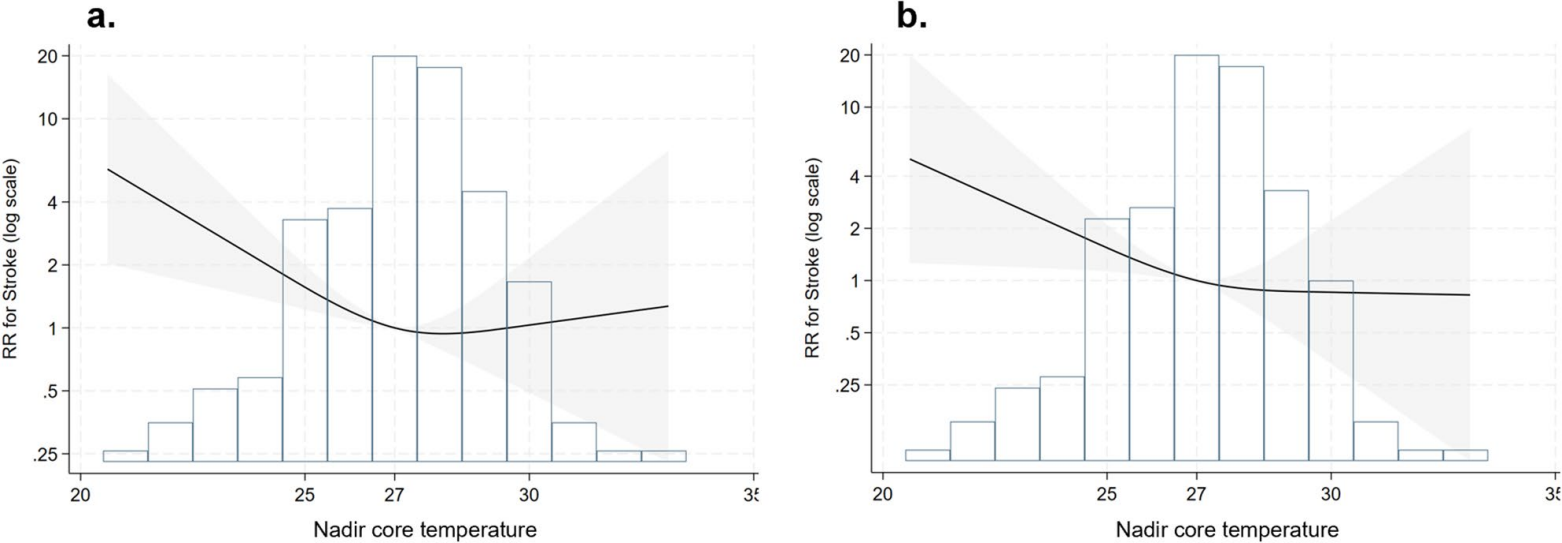
Unadjusted (**a**) and adjusted (**b**) restricted cubic splines demonstrating the risk ratio for strokes based on the nadir core temperature during circulatory arrest. RR, risk ratio

Before adjustment, many variables exceeded the thresholds, indicating an imbalance across groups. For the LMH vs. MH group, while several variables still exceeded the thresholds after adjustment, the overall mean covariate balance improved. For the HMH vs. MH group, most variables met the thresholds after adjustment, with only a few exceptions. Risk ratio (RR) results are presented in Table 4, with the MH group as the reference category. For stroke, the LMH group showed an unadjusted RR of 3.87 and an adjusted RR of 4.46, both statistically significant. Similarly, for bleeding, LMH showed an unadjusted RR of 3.87 and an adjusted RR of 4.05, both statistically significant. In the unadjusted RCS (Fig. 2, a), the lowest risk was observed at the reference point of 27 °C, with a markedly higher RR at lower temperatures and a slight increase at higher temperatures. In the adjusted RCS (Fig. 2, b), a similar increase in RR was observed at lower temperatures, but RR gradually decreased at higher temperatures.

**Table 4:** Modified Poisson regression, using the MH group as the reference

	Group	No adjustments				Adjustment for Stabilized Weight			
		RR	95%CI		*P* value	RR	95%CI		*P* value
30-day mortality	LMH	0.86	0.20	3.78	0.841	1.11	0.19	6.56	0.912
	HMH	0.73	0.32	1.68	0.459	1.12	0.45	2.79	0.812
Stroke	LMH	3.87	1.77	8.44	0.001	4.46	1.72	11.56	0.002
	HMH	1.20	0.59	2.46	0.610	1.78	0.82	3.86	0.142
Newly hemodialysis	LMH	1.72	0.57	5.21	0.339	1.76	0.49	6.31	0.387
	HMH	0.67	0.29	1.57	0.355	0.76	0.31	1.88	0.557
Reoperation for bleeding	LMH	3.87	1.93	7.73	0.000	4.05	1.72	9.53	0.001
	HMH	1.28	0.68	2.43	0.444	1.65	0.82	3.30	0.159
Spinal ischemia	LMH	1.93	0.37	10.08	0.434	1.89	0.33	10.79	0.473
	HMH	0.27	0.05	1.47	0.132	0.44	0.08	2.42	0.342

Adjusted models incorporated propensity scores, including confounders such as age, BMI, sex, comorbidities, and surgical factors

## Discussion

In the 1970s, Griepp and colleagues pioneered profound HCA to enhance organ ischemia outcomes by reducing metabolic demand [13]. Recently, target hypothermic temperatures have tended to increase with the adoption of combined cerebral perfusion strategies, including ACP and RCP [14, 15]. The EACTS/STS guidelines for aortic disease recommend high moderate lower body HCA in combination with cerebral perfusion for hemiarch replacement (class Ⅰ, level C) [4]. In contrast, our institutional strategy for selected hemiarch procedures has been to employ hypothermic circulatory arrest without adjunctive cerebral perfusion. However, good outcomes using this strategy have been reported previously [10]. While cerebral perfusion can help prevent cerebral ischemia and prolong the safe duration of circulatory arrest, the use of ACP carries a risk of embolism and damage to the cerebral vessels during manipulation, particularly in cases of TAAD, due to the fragility of the dissected vessels. On the other hand, RCP results in less than optimal cerebral oxygenation. Against this background, the absence of cerebral perfusion in our cohort provides a unique framework to examine the relationship between hypothermic temperature and neurological outcomes without the confounding influence of active cerebral blood flow augmentation.

Another reason why organ protective strategies have not been established is that the specific origin of temperature has not been clearly defined until now. The 2024 EACTS/STS guidelines introduced a new definition of core temperature in HCA, leading to an increase in reports based on this definition [4]. However, these reports focus on cerebral perfusion, and none have assessed pure hypothermia alone. The reason for this is that the strategy of simple HCA in moderate or mild temperature without cerebral perfusion is very rare. Stone et al. reported the difference between brain and nasopharynx temperatures at the start of circulatory arrest had an error of 2.6 °C, with the difference narrowing more during HCA. On the other hand, the errors in bladder and rectal temperatures were 3.6 °C and 7.4 °C, respectively, and the differences were highly variable and unstable. The narrowing of the deviation of those temperatures from brain temperature during HCA was also smaller compared to nasopharynx temperature [16]. Langenhorst et al. similarly showed a discrepancy between brain temperature and bladder and rectal temperatures [17]. So, it is questionable whether core temperature reflects brain temperature or, in other words, is an indicator of brain protection.

In this study, the lower the nadir core temperature, the more frequent preoperative renal failure and carotid malperfusion were. One possible explanation for the former finding is that bladder temperature is difficult to decrease in the presence of renal failure. The temperature is based on the tympanic temperature at the start of HCA. If the core temperature is not below 30 °C at this point, cooling is extended to reach that target, which may result in the temperature dropping below the target due to inertia after circulation cessation. The latter difference is thought to result from a delayed decrease in tympanic temperature due to reduced cervical blood flow, which extends the cooling time and subsequently results in a lower measured core temperature. In other words, one of the factors associated with lower core temperature may be poor cervical vascular status, which may contribute to an increased risk of postoperative stroke.

The outcomes of this study demonstrated that postoperative reoperation for bleeding and stroke were significantly more frequent in the LMH group than in the MH and HMH groups.

It is an established fact that the coagulation system becomes more disturbed and hemorrhagic the lower the temperature drops during HCA, and an increase in reoperation for bleeding is to be expected [18]. However, Keenan et al. showed that lower temperature during HCA might increase perioperative blood loss and plasma transfusion requirement, although these differences do not translate into increased reoperation for bleeding, a finding that contrasts with our study [19]. Indeed, our rate of reoperation for bleeding, (52/358, 14.5%) was higher than in previous reports. As shown by the preoperative status, our policy is to accept all patients at any time and never refuse an emergency operation, even in people of old age or those in poor condition, except for unsuccessful resuscitation or patient refusal. This approach inevitably results in the inclusion of a higher proportion of high-risk patients, which may have contributed to the observed outcomes.

The high rate of stroke associated with lower nadir core temperature was considered to be largely attributable to preoperative factors, such as malperfusion of the carotid artery, as mentioned above, but it was still significantly higher in the LMH group after adjustment. Adjusted RCS analysis showed the RR of stroke increased when the nadir core temperature during HCA was lower than 27 °C, while the RR remained relatively unchanged at higher temperatures. Seese et al. examined the optimal HCA temperature for elective hemiarch replacement with ACP via RCS. However, their study did not reveal a significant nonlinear relationship between the nadir core temperature and neurological composite outcomes [6]. Similarly, Xue et al. found no correlation between the nadir temperature and stroke undergoing total arch replacement with bilateral ACP, while they concluded that cooling to a temperature of 26 °C and 27 °C was associated with the lowest incidence of stroke in unilateral ACP [7]. These outcomes were similar to those of our study despite the absence of cerebral perfusion. It was thought that the lower the nadir core temperature during HCA, the more brains should be protected, but this was not the case. These findings suggest that reductions in tympanic temperature may be more directly related to cerebral cooling than reductions in core temperature, and that core temperature alone may not adequately reflect cerebral protection.

Other organs, such as the spinal cord, visceral, and kidneys, exhibited no significant difference in postoperative complications based on temperature. Kinoshita et al. demonstrated that cooling, rather than ACP, plays a more critical role in protecting the lower spinal cord during HCA, as ACP did not sufficiently perfuse the lower thoracic cord to affect oxygenation levels [20]. Given this, it is likely that these organs could be protected at MH temperatures during HCA, provided the duration is kept to about 30 min, even when adjunctive cerebral perfusion is not routinely employed.

If brain temperature was adequately reduced (tympanic temperature 25 °C in our study), then a decrease in core temperature was not associated with a lower risk of stroke. Instead, nadir core temperatures below 27 °C were associated with an increased risk of stroke. In addition, the risk of hemorrhagic complications increased with decreasing temperature. Based on core temperature, the results suggest that lowering the temperature to MH may be sufficient for organ protection, whereas the harms of lowering the temperature are likely to outweigh the benefits. Seese et al. demonstrated that elective TAR with ACP for aneurysm, with a nadir core temperature of 27 °C, provides the greatest early survival benefit and the lowest risk of postoperative complications [6]. We hypothesize that poor outcomes might be linked to coagulation abnormalities and systemic inflammation associated with excessive hypothermia. Notably, this apparent turning point in core temperature was similar regardless of the use of cerebral perfusion, suggesting that excessively lowering core temperature solely due to the absence of cerebral perfusion may not be necessary. Additional cerebral perfusion may be more effective to avoid lowering core temperature too much when the brain temperature is difficult to cool, in addition to its established role as a cerebral protective strategy. Consequently, because it is best not to extremely reduce core temperature with a reduced brain temperature, it may be essential to monitor and discuss pharyngeal or tympanic temperatures as a surrogate of brain temperatures as well as core temperature.

### Limitations

This study has several limitations. Firstly, this was a retrospective, non-randomized, single-center study, which carries inherent biases. Secondly, although we adjusted for potential confounders using appropriate statistical methods, some imbalance in covariates remained after adjustment. Several variables still exceeded predefined thresholds, indicating residual imbalance. While covariate balance improved overall, it was not fully achieved. In addition, the relatively small number of patients in the LMH group limited the statistical power for that subgroup and may have affected the precision of the estimates. Thirdly, insufficient brain protection may have resulted in transient neurological impairments, postoperative delirium, confusion, agitation, and a reduced level of consciousness, even in the absence of detectable structural abnormalities on imaging. This study could not assess conditions that were not visible on the images. Fourthly, brain temperature was assessed using tympanic temperature. Although esophageal or pharyngeal temperature is considered to better reflect brain temperature, tympanic temperature was used because of its feasibility in emergency settings. Therefore, some discrepancy between the measured and actual brain temperature cannot be excluded. Finally, this study refers to cases with circulatory arrest times of 30 min or less because of additional cerebral perfusion in cases of longer circulatory arrest times. If this is exceeded, the results would probably be different. These results can generate hypotheses. Nevertheless, they should be validated further.

## Conclusion

This study indicated that, when core temperature was used as an indicator, further temperature reduction was not associated with improved cerebral protection; rather, the risk of stroke and reoperation for bleeding increased with decreasing temperature. Therefore, in the discussion of neurological complications, as reported in recent studies, reliance on core temperature alone may be inappropriate in this specific clinical context, particularly when brain temperature is adequately reduced.

## Supplementary Information

Supplementary Material 1.

## Data Availability

No datasets were generated or analysed during the current study.
